# A Review on Advanced Sensing Materials for Agricultural Gas Sensors

**DOI:** 10.3390/s21103423

**Published:** 2021-05-14

**Authors:** Calvin Love, Haleh Nazemi, Eman El-Masri, Kenson Ambrose, Michael S. Freund, Arezoo Emadi

**Affiliations:** 1 Department of Electrical and Computer Engineering, University of Windsor, Windsor, ON N9B 3P4, Canada; lovec@uwindsor.ca (C.L.); nazemih@uwindsor.ca (H.N.); elmasrie@uwindsor.ca (E.E.-M.); Kenson.Ambrose@uwindsor.ca (K.A.); 2 Department of Chemistry, Dalhousie University, Halifax, NS B3H 4R2, Canada; michael.freund@dal.ca

**Keywords:** carbon nano-tube (CNT) sensors, chemiresistive gas sensors, fibre-optic, gas sensors, multi-walled carbon nanotubes (MWCNTs), polymers, sensing materials, volatile organic compound (VOC)

## Abstract

This work is a comprehensive review of sensing materials, which interact with several target gases pertinent to agricultural monitoring applications. Sensing materials which interact with carbon dioxide, water vapor (relative humidity), hydrogen sulfide, ethylene and ethanol are the focus of this work. Performance characteristics such as dynamic range, recovery time, operating temperature, long-term stability and method of deposition are discussed to determine the commercial viability of the sensing materials considered in this work. In addition to the sensing materials, deposition methods are considered to obtain the desired sensing material thickness based on the sensor’s mechanism of operation. Various material classes including metal oxides, conductive polymers and carbon allotropes are included in this review. By implementing multiple sensing materials to detect a single target analyte, the issue of selectivity due to cross sensitivity can be mitigated. For this reason, where possible, it is desirable to utilize more than one sensing material to monitor a single target gas. Among those considered in this work, it is observed that PEDOT PSS/graphene and TiO${}_{2}$-coated g-C${}_{3}$N${}_{4}$ NS are best suited for CO${}_{2}$ detection, given their wide dynamic range and modest operating temperature. To monitor the presence of ethylene, BMIM-NTf${}_{2}$, SWCNTs and PtTiO${}_{2}$ offer a dynamic range most suitable for the application and require no active heating. Due to the wide dynamic range offered by SiO${}_{2}$/Si nanowires, this material is best suited for the detection of ethanol; a gas artificially introduced to prolong the shelf life of the harvested crop. Finally, among all other sensing materials investigated, it observed that both SWCNTs and CNTs/SnO${}_{2}$/CuO are most suitable for H${}_{2}$S detection in the given application.

## 1. Introduction

A significant proportion of agricultural fruits and vegetables originates from greenhouses, with increasing frequency and a steady rise in harvest land area for crops produced via greenhouse methods [1]. One main reason for the significance of greenhouse practices is that they offer the advantage of year-round production, enabling financial stability for the grower [2]. During the phases of plant growth and storage in agricultural greenhouse environments, there are various volatiles that can affect growth quality and prolong the shelf life of crops, including ethylene, carbon dioxide (CO${}_{2}$), hydrogen sulfide (H${}_{2}$S), ethanol and water vapor (humidity) [3,4,5].

The primary role of CO${}_{2}$ in plants is that it is an essential component of photosynthesis and increases plant productivity by improving growth and vigor [6]. With large qualities of plants undergoing photosynthesis in agricultural environments, CO${}_{2}$ levels in greenhouses can be reduced to less than half of the concentration in some natural outdoor atmospheres [7]. To improve the efficiency of photosynthesis, modern greenhouses artificially inject CO${}_{2}$-air mixtures into the growing environment through the process of carbon dioxide supplementation, otherwise known as “carbon dioxide enrichment”. It is important to regulate the amount of supplied gas within narrow limits, since most crops have a CO${}_{2}$ saturation concentration of typically around 1000 ppm, and levels exceeding 1500 ppm may hinder crop yield [7,8]. The recommended CO${}_{2}$ concentration range for optimal plant growth in a commercial greenhouse operation is 200–1300 ppm [9]. While there are many studies on the impact of CO${}_{2}$ on plant growth, one interesting study by Shimono et al. showed that dissolved CO${}_{2}$ (resulting from high CO${}_{2}$ levels) in solutions surrounding roots affects the plants’ ability to absorb CO${}_{2}$ and where it travels within the plant [10]. Relative humidity is directly related to plant transpiration, and excessive humidity can affect plants by favoring the formation of a Botrytis cinereal infection in mature grape berries, as shown in a study reported by Rossi et al. [11,12]. Low humidity environments for O. sativa have been shown to cause low leaf conductance and CO${}_{2}$ assimilation [5]. For humidity sensors used in greenhouse applications, a detection range of 40–100%, typical of a commercial greenhouse, is ideal [9]. Sulfur is used up in complex metabolic reactions, which produce essential metabolites in plant life. It can be utilized in various forms and taken up in the form of H${}_{2}$S [13]. The effect of H${}_{2}$S on the post-harvest physiology of fruits and vegetables was investigated by Sajid et al., reporting an effective range of 1–80 ppm [14].

In a different perspective, volatiles such as ethanol are known to be beneficial factors in terms of storage. For instance, tomatoes are classified as a perishable fruit and generally have a shelf-life of about 8 days; therefore, a postharvest loss can be a major issue [4]. The dynamic range of common greenhouse vegetables to ethanol exposure for prolonging shelf life is reported to be 500 to 2500 ppm depending on the species [4,15,16]. Ripening and senescence can be delayed using preservatives such as ethanol, which has no detrimental health effect to the crop [4,17]. Ethylene plays a key role in the growth and development of plants as a ripening hormone [3,18]. According to Khan et al., ethylene triggers the network of signaling pathways and influences via interaction with several processes, which are regulated by phytohormones [18]. Moreover, crop adaptability and performance are also influenced by ethylene, under stress conditions. Controlled atmosphere storage tests with ethylene concentrations ranging from 0.001–10 ppm have proven to be successful at prolonging the storage life of commercial produce for several weeks depending on the species [18,19]. The significance of these target analytes in agricultural greenhouses and storage environments is summarized in Table 1.

Because of the key roles played by these small molecules, it is important to monitor them in plant growth environments such as greenhouses, as well as in storage and transport environments. Monitoring of such volatiles can be achieved with sensors that typically require the use of a sensing material. According to B. Eggins, there are three general classifications of sensors containing sensing materials: (1) chemical sensors where the analyte interacts with the sensing material via chemical or physical responses, (2) physical sensors which measure a physical change such as length, weight and temperature and (3) biosensors, which utilize a biosensing element to measure chemical substances [20]. The chemical sensing technologies (including electrochemical sensors) often work by transforming gas concentrations into an electrical signal such as current (amperometric sensors), potential (voltammetric), resistance (chemoresistive sensors) and frequency response (capacitive sensors, acoustic sensors and thermal magnetic) [21,22,23].

Among the common types of physical sensors are mass or gravimetric sensors, which measure changes in resonant frequency due to a mass imposed by an analyte and commonly employ the use of quartz-crystal microbalances (QCMs) [24]. In addition, a recent review on capacitive and piezoelectric-based gas sensors, which operate based on resonant frequency, was published by Nazemi, et al. [25]. In the aforementioned manuscript, new applicatiosn of capacitive micromachined ultrasonic transducer (CMUT) and piezoelectric micromachined ultrasonic transducer (PMUT) in gas sensing technology, are explained, which correlate target gas concentration to resonant frequency shift.

Some commonly reported classes of sensing materials are metal oxides [26,27], polymers (conducting and non-conducting) [28,29], and carbon nanotubes (including other allotropes of carbon such as graphene) [30,31]. There are also reports of multiple classes used simultaneously, like metal oxide/CNTs composites [32], polymer/graphene composites [33] and less commonly used materials like metal-organic frameworks (MOFs) [34] and ionic liquids [35]. This review discusses the above-mentioned types of sensing materials in terms of their method of application onto a sensor, physical properties once applied and mechanism of operation for detecting relevant agricultural analytes such as ethylene, CO${}_{2}$, ethanol, H${}_{2}$S and water vapor. It is observed that some of these sensing materials demonstrate sensitivity and selectivity to certain target analytes, while others are responsive to multiple analytes present in an agricultural greenhouse environment.

Various methods for applying such sensing materials are investigated and described, including drop-coating, spray-coating, dip-coating, and inkjet printing, which vary in complexity and effectiveness for batch fabrication of gas sensors. The sensing materials described in this work were experimentally tested to determine their dynamic range, long term stability and cross sensitivity to interfering gases. A common theme for experimental testing involved a testing chamber, containing a sensor coated with the sensing material, an analyte source and instrumentation required for detecting sensor response to the target gas. The experimental setup used for each sensing material is dependent on the sensing mechanism in which the material is deposited. Amongst the investigated sensing systems, some operate at room temperature, which is ideal for greenhouse applications, while others work at elevated temperatures and require active heating. Moreover, the scrutinized sensing technologies include systems that demonstrate a low limit of detection, as well as the ones that offer a wide detection range. These features are reported to be dependent on the thickness of the sensing layer and sensing technology. This demonstrates the tunability of potential detectors in terms of their sensitivity to the target analyte to meet the needs of the application; be it prolonging shelf life or monitoring the health of the greenhouse crop.

## 2. Sensing Materials for Target Analyte Detection

Many sensing materials have been developed for the detection of gaseous analytes over the past decade [36,37]. Some of these reports demonstrate sensing materials which use expensive starting materials and are complicated to synthesize, which hinders there usefulness in producing a commercially viable gas detector. On the other hand, other reports demonstrate simplicity in sensing material synthesis and their deposition simplicity [38,39,40]. In general, these sensing materials exhibit weak, reversible physical interactions with analytes during exposure, resulting in adsorption or absorption [40,41]. Articles which do not report a significant proportion of the parameters listed in Table 2 were excluded from this review. The sensing materials included in this section were selected based on the completeness of the relevant performance characteristics and their feasibility as detectors for the target analytes, which are the focus of this work.

Typically, a sensing material employed as a gas sensor will react with multiple gases, and for this reason, multiple sensing materials are employed in a network of gas sensors known as an electronic nose (eNose) system. By recording the response of this network of gas sensors, a signature which relates to the target analyte is detected, mitigating the issue of selectivity. For this approach to work however, multiple sensing materials are required which react to a single target analyte. This section discusses recent reports of a diverse range of sensing materials developed for the potential use in gas sensor fabrication for commercial greenhouse and agricultural storage applications. While deposition techniques and sensing mechanisms do play a vital role in the performance of the device, the focus of this work is on the performance characteristics of the sensing materials, including operating temperature, sensitivity, material composition and dynamic range. Where possible, cross sensitivity to interfering gases, temperature dependency, RH susceptibility and long term stability are noted for the sensing materials in this section.

### 2.1. Ethylene Detection

Ethylene gas in the agricultural setting is referred to as a plant hormone which plays a crucial role in many phases of plant biology, such as seed germination, flowering and fruit ripening [18]. These factors demonstrate the importance of ethylene gas detection and monitoring in an agricultural greenhouse setting and during long-distance transportation when unripe fruits can artificially ripen and even overripen during storage. Ethylene atmospheric concentration levels are typically 0.001–10 ppm in food storage and transportation settings, thereby defining the dynamic range of a viable commercial ethylene gas detector [19,42].

Many metal oxide materials have been developed and tested for the detection of ethylene. Li, Jin et al. reported the use of porous zinc oxide nanosheets (ZnO NS) as an ethylene sensing material to determine fruit ripeness [43]. Film preparation occurred via a wet-chemical reaction of zinc acetate with urea followed by annealing at 500 °C. Ethylene detection was possible with a sensitivity of 0.5 µA/ppm and a limit of detection of 5 ppm. This material exhibits a weak sensitivity to VOCs such as benzene, chloroform and chlorobenzene; sensitivity response for ethylene was increased with elevated temperatures (350 °C–500 °C). A long term stability study on the ZnO NS, which was tracked for 30 consecutive days, showed no appreciable change in the materials response to ethylene [43]. Another notable metal oxide; commercially available tin oxide, SnO${}_{2}$ nanoparticles, reported by Agarwal and colleagues, show the capability of detecting ethylene at 20 ppm levels with CO${}_{2}$, SO${}_{2}$, NH${}_{3}$, NO${}_{2}$, and H${}_{2}$S, NH as an interfering gas at concentrations ranging from 1000–3000 ppm at room temperature [27]. It is interesting to note that by introducing Pd/Pt nanoparticles, the sensitivity, selectivity and response time were increased by 39%, 66% and 40%, respectively [27]. Among the most suitable sensing materials for ethylene detection in food storage, Zhang et al. used nanoporous platinum titanium-oxide (PtTiO${}_{2}$) as a sensing material, detecting ethylene at levels below 1 ppm, operating at 19 °C and 19% RH [44]. The sensing material was prepared via the hydrolysis and condensation of mixed-alkoxide precursors into a sol-gel. Further experiments show that a platinum coating can enhance the sensing material’s adhesion to the device for better sensing response.

In regards to carbon allotropes, Swager et al. used single-walled carbon nanotubes (SWCNTs) functionalized with 4-pyridyl moieties as a sensing material in monitoring the senescence in red carnation via the detection of trace levels of ethylene gas [45]. Synthesis of this material was performed via solution phase functionalization of the SWCNTs using iodonium salt reactions [46]. This system operated in air at 23% RH under ambient conditions and could detect 500 ppb of ethylene gas. Moreover, there was weak sensitivity toward internal olefins, but no sensitivity toward common volatile organic compounds (VOCs) was observed.

In a different class of viable materials, the use of commercially available ionic liquids (ILs), such as 1-butyl-3-methylimidazolium bis(trifluoromethyl sulfonyl)imide ([BMIM][NTf${}_{2}$]), is reported by Zevenbergen et al. Even though the sole focus was ethylene (no interference experiments with other gases), this sensing material possesses a detection limit of 760 ppb and operates at room temperature in the presence of 60% RH (optimal response) [35].

An exotic sensing material comprising a fluorophore-tagged Grubbs catalyst showed good selectivity towards ethylene amongst other interfering gases such as CO${}_{2}$, SO${}_{2}$, NH${}_{3}$, NO${}_{2}$ and H${}_{2}$S, and interestingly, no selectivity toward common VOCs [47]. This material functions optimally at room temperature and is stable when exposed to air or humidity, therefore sensing applications for ethylene are viable under those conditions. Moreover, in this report, sensing applications involved monitoring ethylene levels (as low as 0.9 ppm) in the ripening of cherries, passion fruits and bananas. This appears to be a novel and exotic sensing material where the well-known first-generation Grubbs catalyst is used in its synthesis via a simple ligand exchange reaction at room temperature [47].

### 2.2. Carbon Dioxide Detection

Artificial CO${}_{2}$ enrichment is among the most popular methods of optimizing the growing environment in greenhouse applications. Concentration levels of 200–1300 ppm are maintained by use of CO${}_{2}$ injection systems to enhance the growing conditions, however concentration levels in excess of 1500 ppm have been shown to hinder crop yield [7,8]. For a CO${}_{2}$ sensing material to be suitable for greenhouse applications, the dynamic range must be within these limits.

A wide variety of metal oxide sensing materials have been reported for their use in CO${}_{2}$ detection. One such sensing material under this class is reported by Karthik et al., who developed a Zinc oxide (ZnO) sensing material, synthesized by the thermal decomposition of precursors such as zinc acetate and zinc nitrate [48]. Though active heating would be required to maintain an operating temperature of 300 °C, this material yields a wide CO${}_{2}$ concentration range and a 50 ppm limit of detection. Another notable metal oxide for use in CO${}_{2}$ detection is cerium oxide (CeO${}_{2}$) nanospheres [49]. Although the CeO${}_{2}$ nanosphere’s cross sensitivity to interfering gases has yet to be investigated, this material provided a decent detection limit (150 ppm) and operated at 100 °C in air and 70% RH. Material synthesis contained a reaction involving cerium nitrate, citric acid and urea under brief stirring and microwave irradiation. An interesting finding from this study was the mass changes incurred by adsorbed CO${}_{2}$ during sensing, i.e., 10.4 mg of CO${}_{2}$ per gram of sensing material, making this material a viable option for low-level mass sensing technologies. Karthik et al. coated a g-C${}_{3}$N${}_{4}$ nanosheet with TiO${}_{2}$, forming a hybrid 2D sensing material for the purposes of CO${}_{2}$ detection [50]. A dynamic range of 100–2500 ppm was demonstrated at room temperature, with a recovery time from 1500 ppm of 35 s [50]. The TiO${}_{2}$ coated nanosheet was fabricated using commercially available materials is viable for the detection of CO${}_{2}$ at room temperature. Though the dynamic range of this material covers the expected greenhouse CO${}_{2}$ concentration levels, a cross sensitivity with H${}_{2}$S has been reported, and for this reason, it may not be suitable for all greenhouse applications [50].

Baltrusaitis et al. reported a material under the polymer class; methylated poly(ethylene) imine (mPEI) for CO${}_{2}$ detection, synthesized by previously reported work [29]. This polymer is also sensitive to sulfur dioxide (SO${}_{2}$) detection, to which the material shows a lower sensory response. The limit of detection for CO${}_{2}$ is 0.011 CO${}_{2}$ volume %. An interesting finding from this study is the added mass of CO${}_{2}$ during sensing experiments, which was approximately 0.188 pg during a specific experiment, and this number is dependent on factors such as the CO${}_{2}$ concentration, exposure time (to the analyte) and the volume of sensing material. Shifting focus to CO${}_{2}$ sensing materials which do not require synthesis within the polymer material class; is the use of graphene and poly(3,4-ethylenedioxythiophene)-poly(styrenesulfonate) (PEDOT/PSS) in synergy [51]. Advantages are that both materials are commercially available and do not require any pre-treatment or further synthesis and good working temperature range (35–65 °C). This sensing material also features a wide dynamic range of 4.7–4500 ppm, making it among the most appropriate CO${}_{2}$ sensing material for greenhouse applications.

Among the viable ionic liquid sensing materials is 1-ethyl-3-methylimidazolium bis (trifluoromethyl-sulfonyl)-imide (EMIM[NTf ${}_{2}$]), which was investigated by Bhide et al. [52]. This material demonstrated selectivity over interfering gases like N${}_{2}$ and O${}_{2}$ when tested at room temperature (up to 200 °C) with 65% RH, and has a detection limit of 400 ppm. This material is more specifically a room-temperature ionic liquid (RTIL) and is commercially available.

Wei et al. reported a rather exotic sensing material, which was a functionalized pillar[5]arene/bipyridine salt for the detection of CO${}_{2}$ at a detection limit of 2.2 ppm [41]. More specifically, the aryl-furanaldehyde functionalized pillar[5]arene was synthesized by a simple 72 h reflux reaction in ethanol using the required reagents and the product, combined with bromodecane bipyridine when used for sensing. This material was investigated in the presence of N${}_{2}$, H${}_{2}$ and O${}_{2}$ as interfering gases (no response observed) and operated at room temperature.

Another class of materials known as metal-organic frameworks (MOF) was reported as a sensing material for CO${}_{2}$ detection [34]. More specifically, MOF UIO-66-ONa was used and demonstrated excellent selectivity for detecting CO${}_{2}$ (at a concentration of 3.5 × 10${}^{7}$ M) among other gases such as CO and NO, when tested at room temperature. One drawback of this report is that this sensing material requires a multistep synthesis. It is interesting to note that CO${}_{2}$ detections were performed on dissolved CO${}_{2}$, which is still viable for agricultural applications.

Another report of mass added during sensing was shown by Lee et al. where aminopropyl-triethoxysilane (ATPES)-functionalized mesoporous silica [53]. This material was also sensitive to humidity (0–80% explored) and operated at ambient temperature. During sensing, about 0.053 pg of CO${}_{2}$ can be adsorbed when exposed to 2% CO${}_{2}$. A study on fibre-optic gas sensors employed in CO${}_{2}$ detection, shows a linear response to CO${}_{2}$ for up to 30% concentration range. In this sensor film, hybrid xerogels are used when tetraoctylammonium hydroxide (TOAOH), 1-hydroxy-3,6,8-pyrenetrisulfonic acid trisodium salt (HPTS, PTS${}^{-}$) and tetraoctylammonium cation (TOA${}^{+}$) phase transfer agent are immobilized within the hybrid xerogels [54]. In this sensor, by increasing CO${}_{2}$ level of concentration, fluorescence intensity of HPTS decreases. In addition, this sensor benefits from hybrid xerogels properties in terms of accurate thickness of material, as well as high gas permeability, reporting a 0.03% limit of detection [54].

### 2.3. Hydrogen Sulfide Detection

Another important analyte worth monitoring in the agricultural setting is hydrogen sulfide (H${}_{2}$S), which is injected into the growing environment as means of delivering sulfur to the crop. A H${}_{2}$S detector utilized in an agricultural setting ought to have a dynamic range spanning 1–80 ppm; the reported effective range for sulfur delivery in typical greenhouse vegetation [14].

Under the metal oxide material class, Li et al. used indium oxide (In${}_{2}$O${}_{3}$) nano-cubes for sensing H${}_{2}$S at room 25 °C and 100 °C [55]. Synthesis of this material involved a cetyltrimethyl ammonium bromide (CTAB)-assisted solvothermal and subsequent calcination process. This material has a very impressive 5 ppb limit of detection and interestingly, selectivity between NO${}_{2}$ (also able to detect) and H${}_{2}$S sensing can be tuned using temperature (25 °C versus 100 °C). Another notable oxide material developed by Phuoc et al.—copper oxide (CuO) coated with carbon nanotubes (CNTs) and tin oxide (SnO${}_{2}$)—demonstrated its usefulness in the detection of H${}_{2}$S detection [32]. This sensing material system is cross-sensitive with NH${}_{3}$, CO and SO${}_{2}$ which had sensory responses of 4.2%, 0.2% and 0.1%, respectively, as compared to 19% for H${}_{2}$S. The operating conditions for this sensor were room temperature, ambient pressure and negligible RH. Phuoc et al. used SnO${}_{2}$ porous nanofibers; another notable metal oxide candidate which features a 1 ppm detection limit in the presence of interfering gases such as SO${}_{2}$, NH${}_{3}$ and CO [56]. Some drawbacks include that the operating temperature range is 200 °C–350 °C and that any RH causes a decrease in sensory response, thus a sensor fabricated from this material would require active heating and RH regulation. Copper oxide/iron oxide heterostructure ordered arrays have been shown by Zhang et al. to detect H${}_{2}$S with 10 ppm limit of detection [57]. The advantage offered by this material is a wide functional temperature range of −15 °C–65 °C at approximately 50% RH. Testing was performed with interfering gases like NH${}_{3}$, methylbenzene and methanol, which showed low sensitivity. A metal oxide, tungsten oxide (WO${}_{3}$), was used in synergy with polypyrroles (PPy) as a hybrid material for detecting H${}_{2}$S [58]. This material was synthesized via the mechanical mixing of WO${}_{3}$ and PPy at various ratios. The limit of detection is 200 ppm, operates at 90 °C and tolerates 60% RH, with background interfering gases like NH${}_{3}$ and NO${}_{x}$. A 2D sensing material for the detection of H${}_{2}$S is developed by Xu et al., using Zn${}_{2}$SnO${}_{4}$ hierarchical quasi-microspheres constructed from nanosheets and octahedral [59]. This material has a higher sensitivity than the previously mentioned materials, with a lower limit of detection of 1 ppb, but requires active heating as its selective response to H${}_{2}$S is achieved at a working temperature of 133 °C [59]. Unlike most other sensing materials, the long-term stability of this material has been documented; showing no appreciable change in sensitivity to H${}_{2}$S tested over a 60-day period [59].

A viable material under the polymer material class—polyaniline/metal chloride nanofiber composites as sensing materials for H${}_{2}$S detection—was reported by Virji et al. [60]. Although only H${}_{2}$S was explored, this material is able to detect 10 ppm and is easily synthesized by solution polymerization of aniline followed by addition of the desired metal chloride. This material requires no active heating, operating at room temperature and is tolerant to elevated RH typically found in greenhouse environments.

Among the carbon allotrope material class, Asad et al. reported single-walled carbon nanotubes (SWCNTs) modified with copper nanoparticles for H${}_{2}$S detection [61]. The SWCNTs possess a synthesis involving acid treatment and sonication. H${}_{2}$S can be detected at 5 ppm and operates at room temperature with tolerance of 40% RH but air and oxygen, however, are absent during most measurements. Moreover, H${}_{2}$, ethanol, acetone and methane are also detectable but with low sensory response.

### 2.4. Ethanol Detection

Like that of ethylene, ethanol plays a major role in preserving the shelf life of perishable fruits and vegetables during storage and transportation. Artificial injection of this plant hormone in the range of 500 to 2500 ppm has been reported to extend the shelf life by 4–6 days depending on the plant species [4,15,16]. An ideal dynamic range for an ethanol detector utilized for agricultural storage and transportation application would cover this reported range.

Among the metal oxide class, palladium/titanium oxide (Pd/TiO${}_{2}$) nanorod arrays and tin sulfide (SnS) nanoflakes were reported by Dutta [62] and Afsar [63]. The Pd/TiO${}_{2}$ nanorod arrays are also sensitive to 2-propanol and able to detect down to 1 ppm of theses alcohol vapors. However, the operating temperature is 100 °C and RH interference was not investigated. The SnS nanoflakes are also sensitive to acetone and 1-butanol, able to detect down to 10 ppm and operate at an optimal temperature of 100 °C as well. Furthermore, both types of sensing materials require high temperature treatment during synthesis, therefore making them less viable/cost effective for use in detectors, compared to the other sensing materials mentioned. An interesting sensing material developed by Shalev utilizes a SiO${}_{2}$/Si Nanowire layer, which offers a wide detection range of 26–2000 ppm [64]. The multiple-gate field-effect transistor (MGFET) sensing mechanism allows for a tunable sensitivity which increases the dynamic range of the device as needed.

Within the polymer material class, Yoon et al. used poly(styrene-co-allyl alcohol) (PSAA) as a sensing material (other materials also shown) in a wireless sensor to detect ethanol, which proved to be cross sensitive to acetone and ethylene [65]. While PSAA did prove to be cross-sensitive with acetone, methanol and ethylene in this work, this polymer is commercially available and has a lower limit of detection of 1150 ppm for ethanol vapor at room temperature. Alfano et al. presented another viable material which contained graphene-like layers, for detecting ethanol and n-butanol [30]. The material is synthesized via the use of nanostructured carbon black through two-step strategy consisting of oxidation/chemical reduction (chemical route) or oxidation/solvothermal reduction (solvothermal route). The preparation complexity of the nanostructured material presented in this work is higher than that of the PSAA mentioned earlier, but has a much lower limit of detection at 50 ppm.

Among ionic liquids, Xu et al. reported a viable material for the detection of ethanol are alkyl-imidazolium halide [66], which can be synthesized using simple solution synthetic methods, or can be procured commercially. Other VOCs such as butanol, toluene, benzene and dichloromethane were also explored, and the lowest detection limit was 6 ppm of VOC vapors when operated at 30 °C; RH interference was also investigated.

### 2.5. Humidity Detection

Humidity detection is among the most important environmental factors concerning greenhouse agriculture, as it contributes to fungal infections such as Botrytis Cineria in grape berries [12] and is directly related to plant transpiration [11]. To fulfill the requirements for a typical greenhouse, an ideal humidity sensor must have a dynamic range of 40–100% RH; the reported humidity range found in commercial greenhouse applications [9].

Within the metal oxide material glass, Zhang et al. reported a graphene oxide/polymer composite for humidity detection [26]. The graphene oxide/polypyrrole (PPy/GO) was synthesized via the solution polymerization of pyrrole then mixing with a GO suspension. There was no investigation of interfering VOCs, however, this material operates at room temperature at a RH range of 11–97%. As a less viable material, due to its synthetic method, copper oxide (CuO) particles were reported by Malook et al. as a sensing material for humidity detection [67]. Although the initial synthesis steps appear relatively simple, the 500 °C calcination step makes this sensing material less desirable for use. However, this material operates at room temperature, like previously mentioned materials and has a detection range of 20–90%. Furthermore, interfering gases such as hydrogen sulfide and ammonia when tested, causes a decrease in response toward humidity.

Shifting focus to the polymer sensing materials, Zhao et al. reported MWCNTs functionalized with poly-L-lysin (PLL) to be a viable sensing material for humidity detection [68]. In this work, the humidity sensing range was 0–91.5% at room temperature, however, no investigation of cross-sensitivity or interfering gases is reported. Synthesis, however, is very simple, which is essentially a 1:1 solution mixture of PLL and MWCNTs, both of which are commercially available. Graphene oxide modified with polyaniline was used as a sensing material for humidity as shown by Wu et al. [33]. The composite was synthesized via a simple chemical oxidative polymerization reaction of polyaniline in the presence of graphene oxide. This material was tested in the presence of carbon dioxide, methane, ethanol, benzene, formaldehyde, and acetone as interfering gases, and operates at room temperature to detect at a range of 20–90% RH. In fibre grating technique, silica/di-ureasil, polyimide (PI) and polyvinyl alcohol (PVA) are used for a range of 0–98% RH, depending on the structure. This group of materials is reported to have a response time between 2 s and 25 min [69,70,71]. In addition to the aforementioned polymer materials, Al${}_{2}$O${}_{3}$${}^{+}$/PSS${}^{-}$ nano film, SiO${}_{2}$ nano-sphere film, PVA, CaCl${}_{2}$ and Poly (ethylene oxide)/ CoCl${}_{2}$ are used as sensing materials in fibre grating techniques, which measure from 20–95% RH with a significantly faster response time of 1 s to 1 min [72]. In evanescent wave monitoring technique, hydrogel, polyacrylic acid (PAA) nanowires, PVA, polyethylene oxide (PEO), ZnO, Ag-Polyaniline, silica/methylene blue, Co/Polyiniline and gelatin are used as sensing materials.

Qi et al. demonstrated a material under the carbon allotrope material class known as chitosan-wrapped multi-walled carbon nanotubes (MWCNTs-CS) for detecting humidity [31]. The MWCNTs-CS sensing material reported in this work showed a high selectivity to RH among interfering vapors such as ammonia, toluene, formaldehyde, ethanol and acetone. Furthermore, the MWCNTs-CS is produced via a relatively simple solution synthesis using a series of commercially available reagents. Operation occurs at room temperature with a sensing range of 11–95%. It is interesting to note that these researchers also reported mass changes during sensing; a 350 ng mass change is observed during a 95% RH sensing experiment, suggesting that MWCNTs-CS may be an acceptable candidate for mass detecting sensing technologies, as well as its current application as a chemiresistor.

Duan et al. reported Halloysite nanotubes as a sensing material for humidity which has a dynamic range of 0–91.5% RH [73]. This material is commercially available and the material preparation involves mixing with deionized water for use and operates at room temperature, making it suitable for applications that require a simplistic material preparation. Another material which appears viable due to its ease of synthesis is molybdenum disulfide nanodiamond (MoS${}_{2}$/ND) nanocomposites, was reported by Yu et al. [74]. This material is synthesized via a series of stirring, sonication, centrifugation and heating steps, starting a molybdate salt, thiourea and ND powder. Humidity detection using this material occurs at room temperature, yielding a dynamic range of 11–97% RH. WS${}_{2}$ nanosheets capable of humidity detection have also been fabricated. Leonardi et al. developed such a 2D sensing material, which demonstrated at sensing range of 8–85% and recovery times of 30 s to 140 s at room temperature [75]. A test of the sensors stability revealed no considerable change in performance after several weeks of testing [75].

For use in fibre-optic sensing applications, a wide range of sensing materials is available for humidity detection. This group of gas sensors employs several techniques, including fibre grating, evanescent wave monitoring, interferometric approach and absorption measurements, as well as hybrid sensors [69,70,76,77]. These materials are used to measure humidity for a range of 1.1–95% RH and response time between 0.5 s and 30 s [70]. PVA, chitosan and tin dioxide are used in an interferometric approach in fibre-optic gas sensors, which are reported to measure a range of 2–98% RH and response time of 0.5 s to 6 s, depending on the sensing material and structural design of the sensor [70]. In hybrid fibre-optic sensors, agarose, PI, PVA, hydrogel and TiO${}_{2}$ are reported to be employed as the sensing materials to measure a range of 20–100% RH and response time of 1 s to 2 s [70]. For absorption fibre-optic techniques, Au-NP/boehmite, ITO, In${}_{2}$O${}_{3}$, PVA, SiO${}_{2}$, CoCl${}_{2}$, xerogel, SiO${}_{2}$ nano particles and polymeric film with Ag nano particles, as the sensing materials with a reported range of 0–100% RH and a response time between 1 s and 2 min [78]. Lastly, for polymer optic fiber sensors (POFs), Leal-Junior et al. utilized poly(methyl methacrylate) (PMMA) for use in humidity detection with a reported detection range of 25–85% RH with a response time of 14 s [77].

## 3. Deposition Methods

One of the challenges in sensor development for agricultural monitoring is to apply the developed sensing material to the active area of a sensor; this is often referred to as material deposition. In addition, since there are different mechanisms of operation for gas sensors due to their different structures, including capacitive and piezoelectric-based, QCM, chemiresistive and fibre-optic gas sensors, particular deposition techniques should be utilized to improve sensors’ performance in static and dynamic operations. Therefore, the sensor’s mechanism of operation, along with desired sensing material thickness and active area, which agree to the optimum sensor’s response point, can define the potential deposition technique. The most common deposition method observed using all the sensing materials mentioned in previous sections, is drop-coating. This technique is mostly used in chemiresistive gas sensors; obtaining a layer of few nanometers sensing material is not required [49,50]. Other common methods that are also suitable and utilized to deposit the aforementioned sensing materials include spin-coating, dip-coating, spraying, electro-spinning, and inkjet printing, as shown in Table 2. Gas sensors such as capacitive-based structures, which have a thick layer of sensing material, can have a negative impact on their operation benefit from the inkjet printing technique [79].

**Table 2:** Material deposition methods, sensing technologies, sensor performance parameters and operating temperatures with various sensing materials and target analytes in gas phase.

Sensing Material	Target Analyte	Sensing Technology	Deposition Method	Material Thickness	Dynamic Range & Limit of Detection	Recovery Time	Operating Temperature	Long-Term Stability	Sensitivity (Output/Input)	Refs.
BMIM-NTf${}_{2}$	Ethylene	Amperometric	Drop-coating	63 µm	760 ppb–10 ppm	-	22 °C	-	51 pA/ppm	[35]
Porous ZnO NS	Ethylene	Chemiresistive	Dip-coating	10 nm	5–2000 ppm	20 s	350–500 °C	30 days	0.6 µA/ppm	[43]
LaFeO${}_{3}$	Ethylene	Chemiresistive	Screen printing	37–38.3 µm	25–5000 ppm	~1 s	20–200 °C	-	0.4 Ω/ppm	[80,81]
SWCNTs	Ethylene	Chemiresistive	-	1 µL	0.5–50 ppm	-	4 °C	16 days	1.2%R/ppm	[45]
SnO${}_{2}$ nanoparticles	Ethylene	Chemicapacitive	Dip-coating/Sputtering	1300 nm	20–100 ppm	~10 s	22 °C	-	0.0531 pF/ppm	[27]
PtTiO${}_{2}$	Ethylene	Magnetoelastic	Dip-Coating	31–155 nm	0.5–50 ppm	-	19 °C	-	8.5 Hz/ppm	[44]
ZnO	CO${}_{2}$	Chemiresistive	Spray pyrolysis	8.3 nm	50–1000 ppm	100 s	300 °C	-	800 Ω/ppm	[48]
PEDOT PSS/graphene	CO${}_{2}$	Chemiresistive	Calibrated spreader	10 µm	4.7–4500 ppm	-	35–65 °C	-	0.004–0.0047%R/%RH	[51]
TiO${}_{2}$ coated g-C${}_{3}$N${}_{4}$ NS	CO${}_{2}$	Chemiresistive	Drop-coating	30 nm	100–2500 ppm	35 s	22 °C	60 days	406 μΩ/ppm	[50]
CeO${}_{2}$	CO${}_{2}$	Chemiresistive	Drop-coating	170–210 nm diam.	150–2400 ppm	~1 s	100–250 °C	-	4.88 kΩ/ppm	[49]
EMIM[NTF${}_{2}$]	CO${}_{2}$	Chemicapacitive	Dip-coating	<1 µm	50,000–1,000,000 ppm	38.5 s	Room temperature	-	29 pF/ppm	[52]
HPTS	CO${}_{2}$	Fibre-Optic	Dip-coating	>1 µm	300–300,000 ppm	50–100 s	22 °C	-	0.00055 a.u./ppm	[54]
mPEI	CO${}_{2}$	Resonator	Spin coating	-	0.011%	-	-	-	8 Hz/ppm	[29]
CuO,Fe${}_{2}$O${}_{3}$	H${}_{2}$S	Amperometric	-	-	10ppm	-	−15 °C–65 °C	-	700 µA/ppm	[57]
CNTs/SnO${}_{2}$/CuO	H${}_{2}$S	Chemiresistive	Spin-coating	>6 nm	10–80 ppm	10 min	25 °C	-	4.41 Ω/ppm	[32]
SnO${}_{2}$ nanofibres	H${}_{2}$S	Chemiresistive	Electro-spinning	150 nm diam.	0.1–1 ppm	230 s	200–350 °C	-	970 kΩ/ppm	[56]
Zn${}_{2}$SnO${}_{4}$ NS	H${}_{2}$S	Chemiresistive	Dip-coating	100 nm	5–1000 ppb	1300 s	133–170 °C	60 days	1.08 MΩ/ppb	[59]
In${}_{2}$O${}_{3}$	H${}_{2}$S	Chemiresistive	Dip-coating	100 um	5 ppb	5 min	25–100 °C	30 days	13.02 kΩ/ppm	[55]
WO${}_{3}$, PPy	H${}_{2}$S	Chemiresistive	-	50–100 nm	200 ppm	>1 day	90 °C	-	490 µV/ppm	[58]
SWCNTs	H${}_{2}$S	Chemiresistive	Spin-coating	1–2 nm diam.	5 ppm–150 ppm	10–15 s	20 °C	-	0.47%R/ppm	[61]
ZnO Nanowires	Ethanol	Chemiresistive	Spin-coating	25 nm diam.	1–200 ppm	120 s	300 °C	-	644 Ω/ppm	[82]
SnS	Ethanol	Chemiresistive	-	-	10 ppm	9 s	200 °C	6 weeks	0.27–13.5%R/ppm	[63]
Pd/TiO${}_{2}$	Ethanol	Chemicapacitive	Nanorod growth	710–750 nm	1–100 ppm	2.4–3.8 s	100 °C	-	7.5%C/ppm	[62]
SiO${}_{2}$/Si NW	Ethanol	MGFET	vapor-liquid-sold growth	16 nm diam.	26–2000 ppm	4 min	60 °C	-	16–40 pA/ppm	[64,83]
PSAA	Ethanol	Resonator	Drop-coating	19.9 nm	13.3 ppm	20 min	24 °C	-	1.5 Hz/ppm	[84]
CuO particles	Water Vapor	Chemiresistive	Drop-coating	140 µm	33–90%RH	-	22 °C	-	0.5– 30 kΩ/%RH	[67]
WS${}_{2}$ NS	Water Vapor	Chemiresistive	Drop-coating	6 nm	8–85%RH	30–140 s	-	several weeks	580 MΩ/%RH	[75]
MWCNTs-CS	Water Vapor	Chemiresistive	-	-	11–95%RH	-	Room temperature	-	2.4 mΩ/%RH	[68]
MWCNTs-PLL	Water Vapor	Chemiresistive	Drop-coating	-	0–91.5%RH	-	Room temperature	-	3.78 kΩ/%RH	[68]
MoS${}_{2}$/ND	Water Vapor	Chemicapacitive	-	-	11–97%RH	-	Room temperature	-	6.5 nF/%RH	[74]
SPEEK	Water Vapor	Impedance-based	Drop-coating	20 µm	11–95%RH	130 s	22 °C	30 days	12– 120 MΩ/%RH	[85]
TiO${}_{2}$ Nanowires	Water Vapor	Impedance-based	Dip-coating	40–50 nm	12–97%RH	<2 min	17–35 °C	250 days	144 kΩ/%RH	[86]
Silica/di-ureasil FBG	Water Vapor	Fibre-Optic	Dip-coating	450–591 µm	5–95%RH	-	5–40 °C	1 year	1.25–7.14 pm/%RH	[87]
PI	Water Vapor	Fibre-Optic	Dip-coating	450–591 µm	5-95%RH	-	−15–20 °C	-	1.85–2.25 pm/%RH	[88]
Al${}_{2}$O${}_{3}$ ${}^{+}$/PSS${}^{-}$ nano-film	Water Vapor	Fibre-Optic	ESA	84nm	22–39%RH	-	24.5 °C	-	1.43 nm/%RH	[89]
SiO${}_{2}$	Water Vapor	Fibre-Optic	ESA	300 nm	20–80%RH	150ms	10–40 °C	-	67.33–451.78 pm/%RH	[90]
CaCl${}_{2}$	Water Vapor	Fibre-Optic	-	3 µm	55–95%RH	-	30 °C	-	1.36 nm/%RH	[91]
CoCl${}_{2}$	Water Vapor	Fibre-Optic	Drop-coating	10 µm	50–95%RH	~40 s	25 °C	-	67–200 pm/%RH	[92]
HEC/PVDF	Water Vapor	Fibre-Optic	Dip-impregnation	-	40–90%RH	-	28 °C	-	0.196 dB/%RH	[93]
PAA Nanowires	Water Vapor	Fibre-Optic	Electrospinning	-	30–95%RH	210 ms	25 °C	-	0.01 dB/%RH	[94]
ZnO Nanorods	Water Vapor	Fibre-Optic	Dip-coating	2.5 µm	10–95%RH	-	25 °C	-	0.0007–0.0057%P/%RH	[95]
PVA	Water Vapor	Fibre-Optic	Dip-coating	8 µm	20–95%RH	500 ms	20–100 °C	7 days	25–980 pm/%RH	[72,96,97,98]
PEO	Water Vapor	Fibre-Optic	Dip-coating	-	85–90%RH	~1 s	22 °C	-	1.17 dB/%RH	[99]
Silica/methylene blue	Water Vapor	Fibre-Optic	Dip-coating	-	1.1-4.1%RH	<30 s	18 °C	-	0.0087 a.u./%RH	[100]
Ag-Polyaniline	Water Vapor	Fibre-Optic	Dip-coating	15–30 nm diam.	5-95%RH	90s	25–30 °C	-	10–29 mV/%RH	[101]
PGA/poly-lysine	Water Vapor	Fibre-Optic	Soaked in polymer	1 µm	50–92.9%RH	5.8 s	-	-	0.01 dBm/%RH	[102]
ZnO	Water Vapor	Fibre-Optic	Dip/Spin-coating	70–80 nm diam.	5–50%RH	35 s	22 °C	-	0.45%dB/%RH	[103]
Co/Polyaniline	Water Vapor	Fibre-Optic	Dip-coating	10.4 µm	20–92%RH	1 min	30 °C	-	0.024–3.406 mV/%RH	[104]
Gelatin	Water Vapor	Fibre-Optic	Dip-coating	80 nm	9–94%RH	~50 s	22 °C	-	0.167 dBm/%RH	[105]
Chitosan	Water Vapor	Fibre-Optic	Dip-coating	-	20–80%RH	-	25 °C	-	81 pm/%RH	[106]

Drop-coating refers to the application of a thin layer of a sample via the deposition of consecutive droplets of a solution to a surface followed by solvent evaporation [107]. Drop-coating is usually performed in a fashion depending on the desired application for the produced film. For instance, the drop-coating of 4-pyridyl-functionalized SWCNTs (conducting material) was performed on a resistance-based sensing device, and therefore, coating of the sensing material was performed until the desired resistance range (1–3 kΩ) was achieved [45]. In contrast, a desired thickness can be obtained using a specific volume and concentration of an analyte, either in solution or dispersed in a solvent. A sensing material thickness of 30 µm was achieved with an ionic liquid, BMIM-FAP (1-hexyl-3-methylimidazolium tris(pentafluoro- ethyl)trifluorophosphate), when 2 µL was drop coated using an Eppendorf precision pipette [35]. It is interesting to note that solely the ionic liquid was used (no additional solvent), which remains in a viscous liquid state, hence an epoxy resin was used for confinement of this sensing material over the device/sensor surface. In the case of solution use, a 19.9 nm thick sensing film was applied to the surface of a sensor using 0.1 µL for drop-coating a 0.5 wt% solution of PSAA [84].

Dip-coating, a seemingly simpler deposition method, has its challenges. Dip-coating essentially involves the immersion of a substrate (sensing device in this case) in a precursor solution (dissolved or dispersed sensing material). This is followed by subsequent vertical lifting from the solution at a certain velocity [108]. As previously mentioned, dip-coating parameters such as solution concentration and vertical lifting speed affect the thickness of the coated films. A limitation of dip-coating is the ability for the sensor to tolerate immersion into solution for the desired period. Lee et al. were able to achieve a sensing material film thickness of 80–150 nm when dip-coating at a speed of 6 mm/min was performed [53]. Li, Fu et al. reported a film thickness of 100 µm using a dip-coating technique from an I${}_{2}$O${}_{3}$ suspension, followed by drying at 120 °C [55].

**Figure 1:**
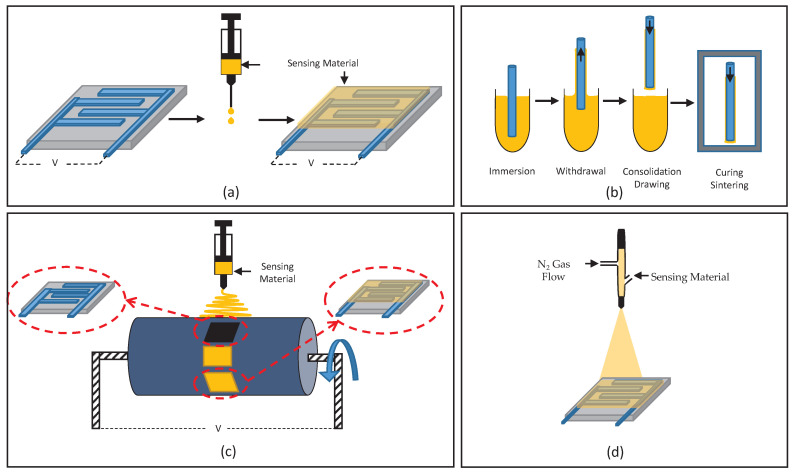
Schematic illustration of deposition methods: (**a**) drop-coating, (**b**) dip-coating, (**c**) electro-spinning and (**d**) spraying.

Another commonly used coating method is spin-coating, where revolutions per minute (RPM) is pivotal in producing the desired film thickness and morphology [29,32,109,110,111]. However, some less commonly used methods, such as spraying [112], inkjet printing [79], in situ oxidative polymerization [113] and layer-by-layer self-assembly [114] have also been reported, some of which are illustrated in Figure 1.

## 4. Conclusions

A comprehensive review of utilized sensing materials in agricultural gas sensors is presented in this work, along with their sensitivity, operating temperature, stability, recovery time, detection range and sensing material thickness. The most common deposition techniques for the sensing materials are also presented. These sensing materials aim to detect carbon dioxide, ethylene, ethanol, humidity and hydrogen sulfide, which are common volatiles involved in crop growth and are controlled in an effort to optimize the growing environment.

As new sensing materials and technologies continue to be developed for use in greenhouse environments, it will be essential to demonstrate their operation in representative environments that explore long-term stability and cross-sensitivity under realistic conditions. The rapid advances in sensing materials, morphology, and structure, as well as transduction mechanisms are expected to address current limitations in performance and are expected to enable miniaturized, low-power sensors capable of achieving wireless, distributed sensor networks for the continuous monitoring of agriculture environments. Further experimentation on the listed sensing materials should be implemented, recording the sensitivity of each material to their respective analyte over a long period of time to validate the usefulness of each material for greenhouse applications. Furthermore, the material’s solubility in water and sensitivity to elevated RH can help determine where the sensor ought to be located within the greenhouse.

### 4.1. Sensing Materials

Greenhouse environments present complex, dynamic, and varied conditions for chemical sensing, making a detailed understanding of cross-reactivity, thermal sensitivity, as well as the effects of water and humidity of various sensing materials and mechanisms critical. While this information is available for sensors such as [BMIM][NTf${}_{2}$], SnO${}_{2}$, PEO, PSAA, PIB, and PMMA, the water solubility to RH is not listed for all sensing materials reported in this review, and further experimentation is necessary.

CNT/SnO${}_{2}$/CuO used for the detection of H${}_{2}$S showed some sensitivity to NH${}_{3}$ and CuO particles revealed a reduction in sensitivity to RH in the presence of H${}_{2}$S and NH${}_{3}$ interfering gases. Sensing approaches for ethylene, CO${}_{2}$, H${}_{2}$S, NH${}_{3}$, ethanol, and humidity suggest that nano sensing materials including nanosheets, nanowires, and nanofibers are capable of sensing low concentrations. However, in addition to thickness and porosity, other properties including density, Poisson’s ratio, Young’s modulus, and saturation level are critical parameters that need to be considered to optimize sensor performance. The impact of these parameters depends on the sensing mechanism, desired sensitivity, and detection range. For example, porous ZnO nanosheets provide a large dynamic range of ethylene detection from 5 ppm to 2000 ppm, suitable for greenhouse detection ranges reported to be 0.001–10 ppm. However, employing [BMIM][NTf${}_{2}$] can provide lower detection limits down to hundreds of ppb, monitoring small releases of ethylene that can result in the over-ripening or spoiling of crops. To monitor ambient CO${}_{2}$ concentration, PEDOT PSS/graphene and TiO${}_{2}$ coated g-C${}_{3}$N${}_{4}$ NS are suitable due to their wide detection range of 4.7 ppm to 4500 ppm and 100–2500 ppm, respectively. SnO${}_{2}$ nanofibers are reported to be useful for a significantly low and limited detection range from 0.1 ppm to 1 ppm for H${}_{2}$S, which is below the natural concentration level in greenhouses. To cover the H${}_{2}$ full target range for greenhouse applications however, CNTs/SnO${}_{2}$/CuO and SWCNTs are most suitable, which offer a dynamic range of 10–80 ppm and 5–150 ppm, respectively, and require no active heating. SiO${}_{2}$/Si NW are reported as a good candidate for ethanol detection in a range of 26–200 ppm which is suitable given the target range of 500–2500 ppm. To detect humidity in greenhouse environments, several viable sensing material options are available. For a wide range of detection between 5% RH and 95% RH ZnO nanorods, Ag-polyaniline, gelatin, PI, and silica/di-ureasil FBG can be potential candidates, considering their fabrication limitations. Taking into consideration the target range for humidity detection, there exist a wide variety of viable sensing materials for greenhouse applications including CuO, SPEEK, TiO${}_{2}$ NW, Silica/di-ureasil FBG, PI, PAA NW, ZnO nanorods, PVA, PEO, AG-Polyaniline, gelatin MWCNTs-CS and MoS${}_{2}$/ND, all of which exhibit a wide dynamic range for humidity detection. Finally, by cycling the operating temperature of ZnO, it may be possible to detect multiple target analytes from a single detector. Although the cross-reactivity from the various ZnO morphologies (porous nanosheets, nanowires and nanorods) has not been reported, this material appears to be the most versatile among other sensing materials reported in this work.

In addition to dynamic range, the limit of detection and sensor fabrication for use in greenhouse applications, some sensing materials operate at ambient temperatures, while others require active heating. This is of particular concern to the grower that wishes to have battery-operated sensors distributed throughout the growing environment. A noticeable trend is that most sensing materials employed in fibre-optic gas sensing can operate at room temperature while many nanofabricated sensing materials such as ZnO nanowires, Zn${}_{2}$SnO${}_{4}$ nanosheets, and TiO${}_{2}$ Nanowires have operating temperatures well above 30 °C. Overall, most of the sensing materials investigated in this work operate at natural greenhouse ambient temperatures. With regards to recovery time, the fibre-optic sensors typically have the lowest recovery times ranging from 0.15–90 s, while the functionalized 2D nanosheet morphologies have reported recovery times of 20–1300 s.

### 4.2. Deposition Techniques

Various deposition techniques can be used to achieve the desired detection ranges of the relevant analytes in agriculture greenhouse environments. Selecting the appropriate deposition method for greenhouse monitoring applications can help achieve the desired thickness and morphology of the sensing material deposited onto the active sensor area. Drop-coating is often a preferred coating method, as it offers a simple, low-cost application of consecutive droplets of solution to a surface followed by solvent evaporation. Therefore, drop-coating can be a potential technique for batch fabrication. For a resistive-based sensing device, drop-coating is recommended, as this deposition method can be performed until the desired resistance range is achieved. It has been observed that the most common deposition method used for fibre optic gas sensors is dip-coating, as shown in Table 2. Dip-coating is a simple method that immerses the substrate in a precursor solution, followed by a vertical removal of the substrate from the solution. However, its limitation is the ability of the sensor to tolerate immersion into solution for the desired period. Spin-coating is another common method where revolutions per minute is used to produce the desired film thickness and morphology. However, with high revolution speeds, it can become difficult for the material to remain on the substrate, making this process less efficient. Although other deposition methods outlined in this review include spraying, layer-by-layer assembly, and inkjet printing, each method consists of its fabrication advantages and challenges.

## Figures and Tables

**Table 1:** Monitoring significance of target analytes in agricultural greenhouse environments.

Target Analyte	Monitoring Significance for Agricultural Greenhouse Environments	Target Range	Refs.
Ethylene	•Ripening hormone which effects the growth and development of plants	0.001–10 ppm	[3,18,19]
	•Influences the crop adaptability and performance under stress conditions		
	•Prolongs the storage life of commercial produce		
Carbon Dioxide	•Essential component of photosynthesis	200–1300 ppm	[6,9]
	•Increases plant productivity by improving growth and vigor		
Hydrogen Sulfide	•Preservative that can delay ripening and senescence of crops during storage	1–80 ppm	[14]
	•Maintains colour and conserves intercellular energy		
Ethanol	•Preservative that can delay ripening and senescence of crops during storage	500–2500 ppm	[4,15,16]
Water Vapor	•Influences leaf conductance and CO${}_{2}$ assimilation	40–100%	[5,9]

## Data Availability

No new data were created or analyzed in this study. Data sharing is not applicable to this article.
